# Analysis of *COQ2* gene in multiple system atrophy

**DOI:** 10.1186/1750-1326-9-44

**Published:** 2014-11-05

**Authors:** Kotaro Ogaki, Shinsuke Fujioka, Michael G Heckman, Sruti Rayaprolu, Alexandra I Soto-Ortolaza, Catherine Labbé, Ronald L Walton, Oswaldo Lorenzo-Betancor, Xue Wang, Yan Asmann, Rosa Rademakers, Neill Graff-Radford, Ryan Uitti, William P Cheshire, Zbigniew K Wszolek, Dennis W Dickson, Owen A Ross

**Affiliations:** Department of Neuroscience, Mayo Clinic, 4500 San Pablo Road, Jacksonville, FL 32224 USA; Department of Neurology, Mayo Clinic, Jacksonville, FL USA; Division of Biomedical Statistics and Informatics, Mayo Clinic, Jacksonville, FL USA

**Keywords:** COQ2, Multiple system atrophy, Genetics, CoQ10 deficiency

## Abstract

**Background:**

Loss of function *COQ2* mutations results in primary CoQ10 deficiency. Recently, recessive mutations of the *COQ2* gene have been identified in two unrelated Japanese families with multiple system atrophy (MSA). It has also been proposed that specific heterozygous variants in the *COQ2* gene may confer susceptibility to sporadic MSA. To assess the frequency of *COQ2* variants in patients with MSA, we sequenced the entire coding region and investigated all exonic copy number variants of the *COQ2* gene in 97 pathologically-confirmed and 58 clinically-diagnosed MSA patients from the United States.

**Results:**

We did not find any homozygous or compound heterozygous pathogenic *COQ2* mutations including deletion or multiplication within our series of MSA patients. In two patients, we identified two heterozygous *COQ2* variants (p.S54W and c.403 + 10G > T) of unknown significance, which were not observed in 360 control subjects. We also identified one heterozygous carrier of a known loss of function p.S146N substitution in a severe MSA-C pathologically-confirmed patient.

**Conclusions:**

The COQ2 p.S146N substitution has been previously reported as a pathogenic mutation in primary CoQ10 deficiency (including infantile multisystem disorder) in a recessive manner. This variant is the third primary CoQ10 deficiency mutation observed in an MSA case (p.R387X and p.R197H). Therefore it is possible that in the heterozygous state it may increase susceptibility to MSA. Further studies, including reassessing family history in patients of primary CoQ10 deficiency for the possible occurrence of MSA, are now warranted to resolve the role of *COQ2* variation in MSA.

**Electronic supplementary material:**

The online version of this article (doi:10.1186/1750-1326-9-44) contains supplementary material, which is available to authorized users.

## Background

Multiple system atrophy (MSA) is a rare sporadic neurodegenerative disorder, with an estimated prevalence ranging from approximately 2–5 cases per 100,000 people [1]. This devastating and rapidly progressive disease is characterized clinically by combinations of poor levodopa responsive parkinsonism, autonomic failure, cerebellar ataxia, and pyramidal symptoms [1, 2]. Widespread presence of glial cytoplasmic inclusions is the neuropathologic hallmark of MSA [3]. To date, there are no effective therapies for MSA and our understanding of the disease etiology remains limited.

Recently, a homozygous mutation p.M128V-V393A/p.M128V-V393A (referred to by Tsuji and colleagues as p.M78V-V343A/p.M78V-V343A) and compound heterozygous mutations p.R387X/p.V393A (referred to by Tsuji and colleagues p.R337X/p.V343A) in the *coenzyme Q2 4-hydroxybenzoate polyprenyltransferase* gene (*COQ2*; OMIM 609825) were identified in affected members of two unrelated Japanese families with MSA [4]. This is the first report of recessive *COQ2* pathogenic mutations in adults, however recessive *COQ2* mutations are known to cause primary coenzyme Q10 (CoQ10) deficiency-1 (COQ10D1; OMIM 607426) which includes phenotype of infantile multisystem disorder or nephrotic syndrome [5–12]; COQ2 is involved in the synthesis of CoQ10. Interestingly, it was also reported that specific heterozygous variation (p.V393A) in the *COQ2* gene may affect susceptibility to MSA [4]. Given these findings, we screened the Mayo Clinic Florida series of 155 MSA patients to assess the frequency of *COQ2* variants in disease. Given the proposed loss-of-function mechanism we also investigated exon dosage as a possible disease-related phenomenon.

## Results

### Result of sanger sequencing and copy number assay

**Figure 1:**
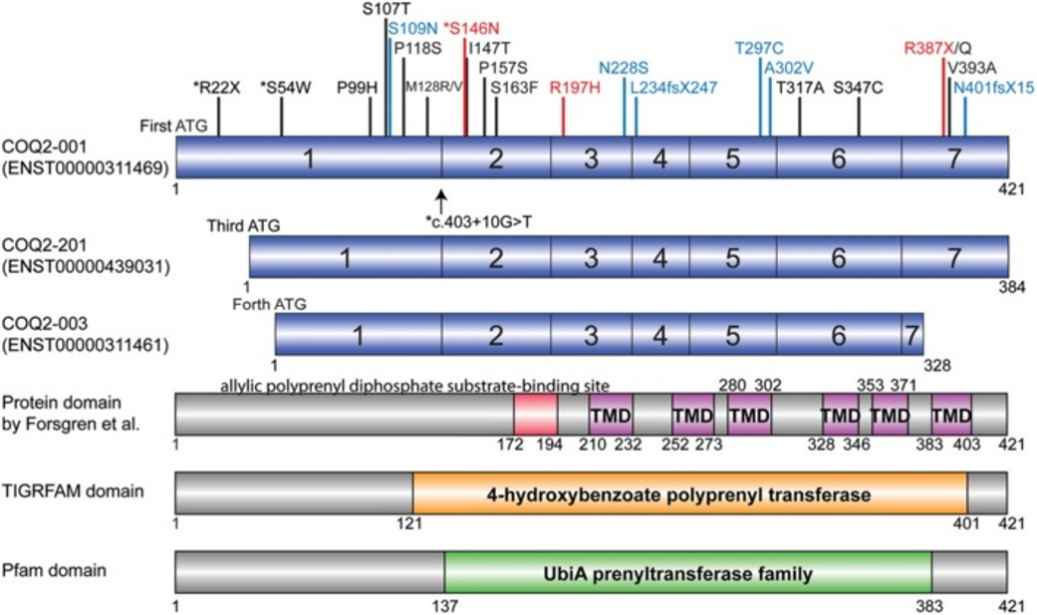
***COQ2*** **variants and transcripts.** The upper three panels in blue represents the protein coding transcripts of the *COQ2* gene and the exons are numbered (transcripts which cause nonsense mediated decay or transcripts which produce no protein are not shown). Transcripts and protein domains are referred to Ensemble (http://useast.ensembl.org/index.html) and 1000 Genomes (http://www.1000genomes.org/home). The lower three panels represent the protein domains and motifs by Forsgren and colleagues [14], TIGRFAMs database (http://www.tigr.org/TIGRFAMs) [15] and Pfam database (http://pfam.xfam.org/) [16]. There were no transcripts which start from second ATG. Red box indicates the allylic polyprenyl diphosphate substrate-binding site. Purple Boxes indicate the six transmembrane domains (TMD) predicted. The orange box and green box show the areas of 4-hydroxybenzoate polyprenyl transferase (Accession: TIGR01474) and UbiA prenyltransferase family (Accession: PF01040) respectively. Potential risk variants and p.R22X shown in black were identified in multiple system atrophy (MSA) [4, 13, 17]. p.R22X only exists on the longest isoform, COQ2-001, and does not associated with MSA. ^†^ENST00000439031 was missing in the latest Ensembl release 77 - October 2014. *Asterisks indicate variants found in our MSA series. Arrow shows an intronic variant, c403 + 10G > T (Exon1 + 10). Mutations in blue were identified in primary coenzyme Q10 (CoQ10) deficiency [5–12]. Mutations in red (p.S146N, p.R197H and p.R387X) were identified both in MSA and primary CoQ10 deficiency [4, 6, 8, 13].

In this study, our sequence analysis of *COQ2* in 155 MSA patients identified eleven variants including one intronic, six synonymous, three non-synonymous and one nonsense variants (Table 1), however exon dosage assays did not detect any deletion/multiplication. Four variants are common, p.R22X, p.L66V, p.D298D and p.S330S, and present in the Exome Variant Server (EVS) and 1000 Genomes; there was no significant minor allele frequency (MAF) difference with our patients, EVS data, or 1000 Genomes data (Table 1; Additional file 1). We found one homozygous and five heterozygous carriers of p.R22X in 155 MSA patients, but there was no significant difference between patients and control subjects or patients and EVS data or 1000 Genomes data (Table 1; Additional file 1). This variant is incorporated into one of the longer *COQ2* transcripts (Figure 1); given the frequency of this variant and the presence of homozygotes both in our study and the EVS, it is unlikely this variant is of clinical relevance in primary CoQ10 deficiency or MSA. Indeed, Schottlaender and Houlden recently reported that p.R22X was more common in controls than cases [13].

**Table 1:** **Variants of** ***COQ2*** **observed in this study**

Exon	Exon 1	Exon 1		Exon 1		Exon 1		Intron 2		Exon 2	Exon 2		Exon 5	Exon 5	Exon 6	Exon 7
rs number	rs183012002	rs112033303		rs376396608		rs6818847					rs121918233		rs199581249	rs6535454	rs1129617	rs141431344
Genotype^a^	c.30G > A	c.64A > T		c.161C > G		c.196G > T		c.403 + 10G > T		c.426A > G	c.437G > A		c.801G > A	c.894 T > C	c.990C > T	c.1107C > T
Amino acid change^b^	p.R10R	p.R22X		p.S54W		p.L66V		Exon1 + 10		p.P142P	p.S146N		p.A267A	p.D298D	p.S330S	p.Y369Y
	Cases	Cases	Cont.	Cases	Cont.	Cases	Cont.	Cases	Cont.	Cases	Cases	Cont.	Cases	Cases	Cases	Cases
Major allele	154	149	339	154	360	87	175	154	360	154	154	360	154	85	89	154
Heterozygous allele	1	5	20	1	0	54	146	1	0	1	1	0	1	64	59	1
Minor allele	0	1	1	0	0	14	39	0	0	0	0	0	0	6	7	0
Total	155	155	360	155	360	155	360	155	360	155	155	360	155	155	155	155
MAF (%)	0.3	2.3	3.1	0.3	0	26.5	31.1	0.3	0	0.3	0.3	0	0.3	24.5	23.5	0.3
OR (95% CI)	NA	0.65 (0.0.21, 1.71)		NA		0.74 (0.50, 1.10)		NA		NA	NA		NA	NA	NA	NA
P-value	NA	0.51		0.30		0.12		0.30		NA	0.30		NA	NA	NA	NA
PolyPhen-2	NA	NA		Benign		Benign		NA		NA	Probably damaging		NA	NA	NA	NA
SIFT	Tolerated	NA		Tolerated		Tolerated		NA		Tolerated	Damaging		Tolerated	Tolerated	Tolerated	Tolerated

^a^cDNA reference (NM_015697.7) from NCBI gene was used to annotate the position of genomic DNA. ^b^Accession number NP_056512.5 from NCBI protein were used to annotate the position of amino acid. P-values result from Fisher’s exact test comparing the frequency of carriers of the minor allele between MSA patients and controls. Estimation of an odds ratio (and 95% confidence interval) is not possible when the rare allele is not observed in one of the comparison groups, and therefore NA is given for these quantities for three of the variants for which the rare allele was observed in MSA patients but not in controls. After applying a Bonferroni adjustment for multiple testing, p-values ≤0.01 (5 tests) were considered as statistically significant. *Abbreviation:* *Cont.* Control subjects, *MAF* minor allele frequency, *NA* not available, *OR* odds ratio, *CI* confidence interval.

Rare heterozygous variants: p.R10R, p.S54W, c.403 + 10G > T (Exon1 + 10), p.P142P, p.S146N, p.A267A and p.Y369Y were observed in one patient each but p.S54W, c.403 + 10G > T (Exon1 + 10) and p.S146N were not found in 360 control subjects. Sequencing for p.R10R, p.P142P, p.A267A, p.D298D, p.S330S and p.Y369Y was not assessed in controls because they are synonymous variants. In total, we identified three *COQ2* variants with unknown significance; p.S54W, c.403 + 10G > T (Exon1 + 10) and p.S146N. The COQ2 p.S146N [6–8] has been shown to affect COQ2 function [7] and was not identified in 500 control chromosomes in a previous report [6].

### Statistical analysis for nine COQ2 mutations found in CoQ10 deficiency-1

Primary CoQ10 deficiency-1 is extremely rare; only 11 patients and nine *COQ2* mutations have been identified so far (Table 2) [5–12]. We investigated the publicly available data (EVS) and these nine variants (p.S109N, p.S146N, p.R197H, p.N228S, p.L234fsX247, p.T297C, p.A302V, p.R387X and p.N401fsX15) were not found in more than 4000 European American subjects. If we perform a meta-analysis of the results from our study, the other case–control studies [4, 13, 17, 18] and EVS data, pathologically-confirmed MSA is associated with *COQ2* mutations previously found in primary CoQ10 deficiency-1 (2/361 vs 0/6356, p value =0.0029, Table 3); clinical MSA did not associate with those nine mutations. (1/1907 vs 0/6356, p value =0.23, Table 3).

**Table 2:** ***COQ2*** **mutations previously found in primary CoQ10 deficiency-1**

#	Amino acid change	Age at onset	Age at death	Clinical phenotype	Reference
1	p.S109N/p.S109N	3 week	5 month	Seizures, hypertrophic cardiomegaly, nephrotic syndrome	[5]
2	p.S146N/p.S146N	5 day	6 month	Nephrotic syndrome, seizures, encephalopathy, hypotonia, respiratory failure	[6]
3	p.S146N/p.S146N	Not-available	Not-available	Not available	[7]
4	p.S146N/p.R387X	birth	2 month	Respiratory failure, lactic acidosis, hypertrophic cardiomyopathy, necrotizing enterocolitis, encepharopathy	[8]
5	p.R197H/p.N228S	18 month	alive at 29 month	Nephrotic syndrome, generalized edema	[6]
6	p.N228S/p.L234fsX247	24 month	Not-available	Nephrotic syndrome, acute renal failure	[9]
7	p.T297C/p.T297C (proband)	12 month	alive at 33 month	Nephrotic syndrome, hypotonia, optic atrophy, psychomotor regression, seizures, hemiplegia	[10]
8	p.T297C/p.T297C (sister)	9 month	Not-available	Nephrotic syndrome	[10]
9	p.A302V/p.A302V (twin, male)	birth	6 month	Respiratory failure, generalized edema, seizures, hypotonia, dystonic-hyper kinetic movement, lactic acidosis, retinopathy	[11]
10	p.A302V/p.A302V (twin, female)	birth	5 month	Respiratory failure, generalized edema, seizures, hypotonia, dystonic-hyper kinetic movement, lactic acidosis, intracranial hemorrhage	[11]
11	p.N401fsX15/p.N401fsX15	birth	12 day	Neurologic distress, liver failure, nephrotic syndrome, pancytopenia, diabetes, cytolysis, seizures, lactic acidosis	[12]

**Table 3:** **Frequency of nine COQ2 mutations found in CoQ10 deficiency-1**

Study	Country	Mutation	Frequency				P value vs. controls		
			Clinical MSA N (%)	Pathologically-confirmed MSA N (%)	Total MSA N (%)	Control N (%)	Clinical MSA	Pathologically-confirmed MSA	Total MSA
This study	USA	p.S146N	0/58 (0)	1/97 (1.0)	1/155 (0.6)	NA	NA	NA	NA
Tsuji et al. [4]	^2^mixed	p.R387X	1/762 (0.1)	0/2 (0.0)	1/764 (0.1)	0/1129 (0)	0.40	NA	0.40
Jeon et al. [17]	Korea	None	0/299 (0)	NA	0/299 (0)	0/365 (0)	1.00	NA	1.00
Sharma et al. [18]	Europe	None	0/788 (0)	NA	0/788 (0)	0/600 (0)	1.00	NA	1.00
Schottlaender et al. [13]	Europe	p.R197H	NA	1/300 (0.3)	1/300 (0)	0/262 (0)	NA	1.00	1.00
^1^EVS	USA	None	NA	NA	NA	0/4000 (0)	NA	NA	NA
Combined	Combined	p.S146N, p.R197H or p.R387X	1/1907 (0.05)	2/361* (0.5)	3/2268 (0.1)	0/6356 (0)	0.23	0.0029	0.019

Total nine *COQ2* mutations previously found in primary CoQ10 deficiency-1, p.S109N, p.S146N, p.R197H, p.N228S, p.L234fsX247, p.T297C, p.A302V, p.R387X and p.N401fsX15, were not found in more than 4000 subjects of EVS. ^1^Data of European American population from Exome Variant Server. ^2^Mixed indicates Japan, Europe and North America. Abbreviation: EVS, Exome Variant Server, MSA, multiple system atrophy, NA, not available. *Thirty eight out of 97 our patients were included in the study of Schottlaender et al. P-values result from Fisher’s exact test. Because only two pathologically-confirmed MSA patients were included in the study by Tsuji et al., we did not make a comparison of pathologically confirmed MSA patients vs. controls for that study.

### In silico analysis

For the c.403 + 10G > T (Exon1 + 10) variant, *in silico* analysis, Human Splicing Finder predicted this variant to change exon 1 splicing site including 8 intronic nucleotides between exon 1 and 2. We performed RNA/cDNA study to validate whether the heterozygous c.403 + 10G > T (Exon1 + 10) caused a splicing effect in the border between exon 1 and 2.

For the COQ2 protein domain, TIGRFAMS domain showed 4-hydroxybenzoate polyprenyl transferase (Accession: TIGR01474) and Pfam database showed UbiA prenyltransferase family (Accession: PF01040) respectively (Figure 1). Main roles of those domains are biosynthesis of cofactors, prosthetic groups, and carriers. We did not find any specific relationship between *COQ2* variants and protein domains (Figure 1), although there is a tendency that the most of variants found in CoQ10 deficiency-1 exist in those of domains found in TIGRFAM and Pfam.

### RNA/cDNA sequencing

RNA/cDNA sequencing showed normal sequence between exon 1 and 2 (after p.R22X) from the cerebellum tissue with homozygous or heterozygous p.R22X (data not shown), suggesting the isoform including first ATG (ENST00000311469) does not play major role in MSA patients (Figure 1). Mitsui and Tsuji reported that the majority of the transcripts in human tissues start near the third or fourth ATG codon [19] and p.R22X lies upstream of the third ATG codon (Figure 1). Analysis of the transcript generation for the carrier of the c.403 + 10G > T (Exon1 + 10) variants did not show any alternate band length and sequencing of the amplified cDNA product did not show any alternate nucleotide inclusion.

### Clinical and pathological presentation of a patient with heterozygous COQ2 p.S146N variant

**Figure 2:**
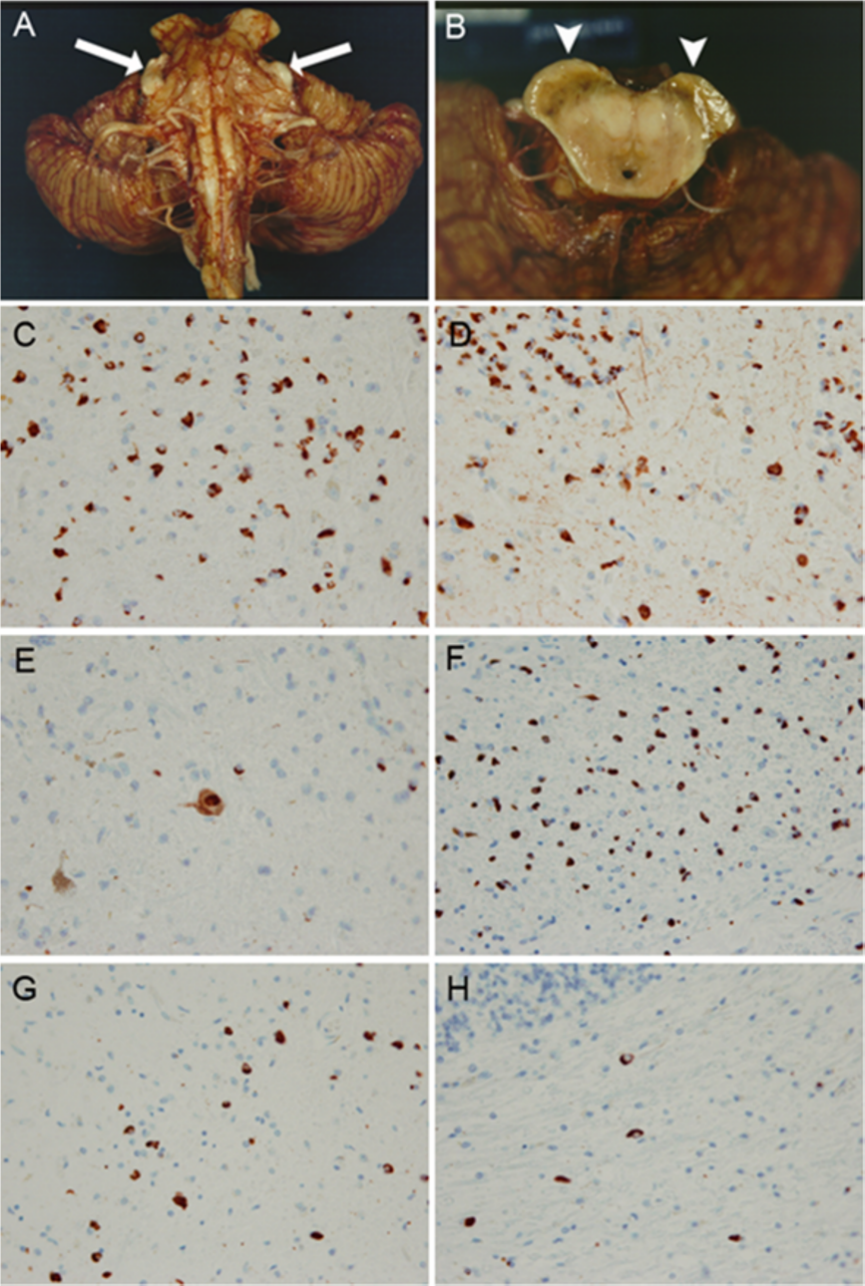
**Macroscopic features and α-synuclein pathology of a patient with heterozygous COQ2 p.S146N variant (A-H).** The infratentorial tissues were remarkable for severe atrophy of the pontine base and the inferior olive **(A)**. The trigeminal nerve was unusually prominent due to the extreme pontine atrophy (arrows). Horizontal sections of the midbrain were remarkable for extremely marked atrophy **(B)**. The substantia nigra had loss of pigmentation. The cerebrum peduncle was severely atrophic (B, arrowheads). Immunohistochemical staining for α-synuclein (LB509, mouse monoclonal; 1:100) in the globuls pallidus **(C)**, putamen **(D)**, substantia nigra **(E)**, pontine nucleus **(F)**, inferior olive **(G)** and the cerebellar white matter **(H)** (magnification × 400).

This patient was a Caucasian male who had been clinically diagnosed with MSA-C (cerebellar variant of MSA) during his life. There is no family history of any similar disorder or other neurological disorders. His initial symptoms were staggering gait and frequent falls at the age of 54 years. A brain MRI showed marked atrophy of the pontine and cerebellar vermis at the age of 55 years. He had deterioration of his coordination, with not only worsening of his gait but also of his limbs and speech difficulties. Autonomic dysfunction included difficulty emptying the bladder, necessitating self-catheterization. Other autonomic dysfunction was otherwise suggested by several syncopal episodes. He was wheelchair bound at the age of 60 years. Muscle strength was normal. Deep tendon reflex were abnormally brisk at all sites and bilateral Babinski sign was positive. Severe ataxia was seen on finger to nose to finger test and heel-knee-shin testing. His treatment was limited to speech and physical therapy. He died of septicemia at the age of 68 years.He had an autopsy examination (Figure 2). The fixed tissue of the case weighted 680 grams and the calculated brain weight was 1360 grams. The infratentorial tissues were remarkable for severe atrophy of the pontine base and the inferior olive (Figure 2A). Horizontal sections of the midbrain, pons and medullar were remarkable for extremely marked atrophy. The substantia nigra had loss of pigmentation. The cerebrum peduncle was severely atrophic (Figure 2B, arrowheads). Microscopic analysis showed the neocortex was unremarkable in most areas. The pontine base and the inferior olive had severe neuronal loss and gliosis. In addition there were GCIs (Figure 2F & G). The cerebellum had severe diffuse loss of Purkinje cells with many axonal torpedoes. The internal granular cell layer had diffuse depopulation. The deep cerebellar white matter was markedly attenuated and gliotic with GCIs (Figure 2H). The pathological findings were of MSA (striatonigral and olivopontocerebellar degeneration), though the degree of the α-synuclein pathology was more severe than for typical MSA cases.

## Discussion

Our study examined the relevance of *COQ2* variation (and subsequent CoQ10 deficiency) in susceptibility to MSA. The absence of pathogenic recessive *COQ2* mutations or copy number variants within our series of clinical and pathologically-confirmed MSA patients means further screening of case–control series and functional studies are likely required to confirm the role of this candidate gene in MSA. We identified two heterozygous *COQ2* variant, p.S54W and c.403 + 10G > T, of unknown significance, although cDNA study for c.403 + 10G > T did not show any alternate splicing. Recently, three independent case–control studies also reported an absence of recessive *COQ2* mutations in MSA supporting the rarity of this as a potential cause of disease [13, 17, 18]. In addition, Jeon and colleagues noted the Asian-specific genetic risk factor p.V393A was observed at the same frequency in cases and controls (~2.6%). These reports question the role of *COQ2* variants in risk of MSA but do not rule out the potential for rare variants in disease.

Interestingly, we found one heterozygous COQ2 p.S146N variant. Clinical and pathological presentation of our Caucasian patient harboring the heterozygous p.S146N variant was MSA-C with severe pathological changes. This mutation has been previously reported as a pathogenic homozygous mutation in infantile multisystem disorder (a major phenotype of primary CoQ10 deficiency-1) [6, 7] or as part of a compound heterozygous state [8] (Table 2). The pathogenicity of this mutation is supported by the high conservation at this amino acid position, by the prediction as probably damaging by PolyPhen-2 and SIFT, and by the finding of abnormal mitochondria proliferation and low CoQ10 levels in the skeletal muscle of carriers [6].

The study of Bujan and colleagues demonstrated that a patient carrying the homozygous p.S146N mutation showed significant CoQ10 deficiency in fibroblasts and presented deficient CoQ10 biosynthesis [7]. Collectively, these reports suggest that the p.S146N substitution functionally impairs CoQ10 activity and therefore it is possible that in the heterozygous state p.S146N might increase susceptibility to MSA. Our patient with heterozygous p.S146N was clinically diagnosed as MSA-C. Fitting with this, it was reported that patients with *COQ2* mutations have increased frequency of MSA-C compared to MSA-P (the parkinsonian variant of MSA) and the cerebellum is more vulnerable to compromised COQ2 function than other regions of the central nervous system [4]. A previous study has also revealed that the cerebellum in both rats and humans contains the lowest concentration of CoQ10 in the brain [20]. Recessive *COQ2* mutations are known to cause primary CoQ10 deficiency-1, including infantile multisystem disorder, it remains unresolved why the same gene would cause MSA in an adult [5–12]. One possible explanation would be that the decrease in COQ2 activity associated with the recessive mutations in patients with MSA is milder than that observed in patients with primary CoQ10 deficiency-1 [4].

This report is the third time a mutation known to cause primary CoQ10 deficiency-1 has been observed in an MSA case. Recently Tsuji and colleagues found R387X (referred to R337X by Tsuji et al.) in one of the original multiple families (Family 12) and Schottlaender and Houlden (MSA Brain Bank Collaboration) reported one heterozygous p.R197H carrier in 300 pathologically-confirmed MSA [13]. *COQ2* mutations found in CoQ10 deficiency-1 were associated with MSA (3/2268 vs 0/6356, p value =0.019, Table 3), especially pathologically-diagnosed MSA (p value =0.0029). Although primary CoQ10 deficiency-1 due to *COQ2* mutations is rare, it may be worth reassessing family history in these patients for the possible increased occurrence of MSA.

Previously, several genetic risk factors for MSA have been reported. For instance, a common variant in the α-synuclein gene (*SNCA* rs11931074) was associated with MSA in an initial study (Odds ratio [OR] = 6.2, 95% confidence interval [CI]: 3.4 – 11.2, p value under recessive model =5.5 × 10^−12^) [21] and this result was subsequently replicated in an independent set of pathologically-confirmed MSA cases (OR: 4.7, 95% CI: 1.0 – 21.7, p value =0.06) [22]. Another common variant, the microtubule-associated protein tau (*MAPT*) H1 haplotype (rs1052553) has also been associated with risk of MSA (OR: 1.9, 95% CI: 1.1 – 3.2, p *=*0.016) [23]. On the other hand, rare variants (MAF < 1%) have not been reported to be associated with MSA, probably due to the low disease frequency of MSA. Copy number loss of “src homology 2 domain containing-transforming protein 2 gene” was reported as a risk of MSA [24] but a replication study from another institute failed to show the association [25]. Further studies of rare variants and copy number variation are warranted to elucidate further genetic risks for MSA.

## Conclusions

MSA due to recessive *COQ2* mutations (including exon dosage) was not observed in our study. We did detect the presence of a single heterozygous pathogenic *COQ2* variant, which causes infantile multisystem disorder (primary CoQ10 deficiency-1) in a homozygous condition. Given the lack of therapeutic options in MSA, more work is warranted to resolve the role, if any, of *COQ2* variants in disease. Primary CoQ10 deficiencies, with early treatment, respond well to CoQ10 supplementation [26]. Whether this is a viable option for treatment or prevention of MSA, or within in a small subset of individuals carrying *COQ2* mutations, remains to be determined. Further studies on the etiology of MSA will lead to more rational and improved drug discovery.

## Methods

### Subjects

A total of 97 pathologically-confirmed MSA patients, 58 clinically-diagnosed MSA patients, and 360 control subjects from the United States were included in this study (Table 4). Thirty eight pathologically-confirmed MSA cases were included in a previous study as part of the MSA brain collaboration study [13]. The diagnosis of MSA was made using current consensus criteria [2]. In MSA patients, mean age was 61.6 ± 9.1 years, and 93 patients (60%) were male. Mean age at evaluation in controls was 70.0 ± 13.4 years, and 142 control subjects (39.4%) were male. All control individuals were free of personal or familial history suggestive of parkinsonism, cerebellar ataxia or autonomic failure. All individuals were unrelated within and between sample groups. The Mayo Clinic Institutional Review Board approved the study, and all living subjects provided informed consent. Informed consent for pathologically-confirmed cases was obtained from the next-of-kin.

**Table 4:** **The demographic, pathologic and clinical details of the MSA patients**

	Mayo clinic MSA series		
	Pathologically-confirmed MSA	Clinically- diagnosed MSA	Total
Total number	97	58	155
Male/Female	59/38	34/24	93/62
Positive family history of MSA	0	2	2
Positive family history of PD	1	10	11
Mean age at onset, SD	59.1 ± 8.7 (39–78)	64.7 ± 8.8 (48–83)	61.6 ± 9.1 (39–83)
Mean age at death, SD	67.5, 8.5 (47–86)	NA	NA
MSA-P/MSA-C/MSA-PC/Unclassified*	36/13/47/1	49/9/0/0	85/22/47/1
Ethnicity (Race), %			
White (Caucasian)	93	47	140
African-American	1	4	5
Asian	2	0	2
Hispanic	1	0	1
More than One Race/NA	0	7	7

*Abbreviation:* *MSA-P* parkinsonian variant of multiple system atrophy, *MSA-C* cerebellar variant of multiple system atrophy, *MSA-PC* mixed type of multiple system atrophy with parkinsonism and cerebellar ataxia, *NA* not available, *PD* Parkinson’s disease, *SD* standard deviation. *One MSA patient was unclassified.

### Genetic analysis

For direct sequence analysis, each exon was amplified by polymerase chain reaction (PCR) using published primers for *COQ2* [4]. DNA was extracted from frozen brain or whole blood samples using standard protocols. Electropherograms were analyzed with SeqScape v2.5 using 3730 DNA Analyzer (ABI, Applied Biosystems, Foster City, CA, USA). Specific genetic variants observed (p.R22X, p.S54W, p.L66V, Exon1 + 10 and p.S146N) on exon 1 and 2 were screened through a series of US Caucasian healthy control subjects (n = 360) but synonymous (silent) variants were not screened. Of note, *COQ2* gene has multiple start codons and a number of alternative isoforms are expressed. Herein, we have used the consensus RefSeq accession NM_015697.7 to number all variants within the *COQ2* gene and protein; however this does not necessarily indicate this long isoform is the one related to the MSA or primary CoQ10 deficiency phenotype.

Exonic deletion or multiplication of *COQ2* gene was assessed in all patients with MSA. To determine exon dosage genomic copy number probes were used for each individual exon (n = 7; Applied Biosystems) and details are available on request. TaqMan Copy Number Reference Assay RNase P (human) was used as an endogenous control (ABI). Quantitative PCR was carried out using TaqMan expression chemistry protocol as previously described [27]. All assays were performed in triplicate on the ABI 7900HT Fast Real-Time PCR System and analyzed with ABI SDS 2.2.2 Software (ABI).

### RNA/cDNA sequencing

RNA was extracted from brain tissue (cerebellum) using standard protocol with TRIzol reagent (Life Technologies, Foster City, CA, USA). For carriers of specific variation, p.R22X and c.403 + 10G > T (Exon1 + 10), cDNA was made by using High-Capacity cDNA Reverse Transcription Kit (ABI) and sequencing was performed. Primers for cDNA sequencing for the border between *COQ2* exon 1 and 2 are 5'-CACGGTGGTGACTTGCAG-3' (Forward primer sequence) and 5'-TGCTCCACGCATCAGAATAG-3' (Reverse primer sequence).

### In-silico analyses

For prediction of the functional consequences of variants on COQ2 protein sequence, we used software programs available on the internet, namely PolyPhen-2 (http://genetics.bwh.harvard.edu/pph2/) [28] and SIFT (http://sift.jcvi.org/) [29].

The results were shown in Table 1. Splice site prediction of intronic variants identified in *COQ2* were calculated by using the online bioinformatics tools, Human Splicing Finder (version 2.4.1) [30]. See the URL. (http://www.umd.be/HSF/HSF.html).

The protein domain search tools, TIGRFAMs database (http://www.tigr.org/TIGRFAMs) [15] and Pfam database (http://pfam.xfam.org/) [16] were used to elucidate the association between variants and protein domains (Figure 1). We used an online software of DOG (Domain Graph, version 2.0.1; http://dog.biocuckoo.org/) to prepare publication-quality figures of protein domain structures [31].

### Statistical analysis

For each *COQ2* mutation, comparisons of the frequency of carriers of the minor allele between MSA patients and (1) controls from our study, (2) EVS data, and (3) 1000 Genomes data were made using Fisher’s exact test. We utilized a Bonferroni correction to adjust for multiple testing separately for these three sets of comparisons, after which p-values ≤0.01 (MSA patients vs. controls, 5 tests), ≤0.0071 (MSA patients vs. EVS data, 7 tests), and ≤0.0071 (MSA patients vs. 1000 Genomes data, 7 tests) were considered as statistically significant. Note that only *COQ2* variants for which information was obtained for both MSA patients and the given comparison group of interest were utilized when making comparisons. Additionally, when combining data from our study and previously published or publicly available data, we compared the frequency of carriers of any *COQ2* mutation found in primary CoQ10 deficiency-1 (p.S109N, p.S146N, p.R197H, p.N228S, p.L234fsX247, p.T297C, p.A302V p.R387X, and p.N401fsX15; Table 2) [5–12] between MSA patient and control groups using Fisher’s exact test (Table 3). All statistical analysis was performed using R Statistical Software (version 2.14.0; R Foundation for Statistical Computing, Vienna, Austria).

## Electronic supplementary material

Additional file 1: **For variants of** ***COQ2*** **observed in this study, comparisons of MSA patients with controls, with EVS data, and with 1000 Genomes data.** (DOCX 21 KB)

## Abbreviations

COQ2: Coenzyme Q2 4-hydroxybenzoate polyprenyltransferase
CoQ10: Coenzyme Q10
EVS: Exome Variant Server
MAF: Minor allele frequency
MSA: Multiple system atrophy
PD: Parkinson’s disease.
