# Supplementary material for: Analysis of COQ2 gene in multiple system atrophy
Source: Mol Neurodegener. 2014 Nov 5;9:44. doi: 10.1186/1750-1326-9-44 (PMC4233093; doi:10.1186/1750-1326-9-44)
Supplement: Supplementary file 1 — Additional file 1: For variants of COQ2 observed in this study, comparisons of MSA patients with controls, with EVS data, and with 1000 Genomes data. (DOCX 21 KB) [file 13024_2014_554_MOESM1_ESM.docx]

## Additional File 1 - For variants of *COQ2* observed in this study, comparisons of MSA patients with controls, with EVS data, and with 1000 Genomes data

| Exon | Exon 1 | Exon 1 | | Exon 1 | | Exon 1 | | Intron 2 | | Exon 2 | Exon 2 | | Exon 5 | Exon 5 | Exon 6 | Exon 7 |
| --- | --- | --- | --- | --- | --- | --- | --- | --- | --- | --- | --- | --- | --- | --- | --- | --- |
| rs number | rs183012002 | rs112033303 | | rs376396608 | | rs6818847 | |  | |  | rs121918233 | | rs199581249 | rs6535454 | rs1129617 | rs141431344 |
| Genotype^a^ | c.30G>A | c.64A>T | | c.161C>G | | c.196G>T | | c.403+10G>T | | c.426A>G | c.437G>A | | c.801G>A | c.894T>C | c.990C>T | c.1107C>T |
| Amino acid change^b^ | p.R10R | p.R22X | | p.S54W | | p.L66V | | Exon1+10 | | p.P142P | p.S146N | | p.A267A | p.D298D | p.S330S | p.Y369Y |
|  | Cases | Cases | Cont. | Cases | Cont. | Cases | Cont. | Cases | Cont. | Cases | Cases | Cont. | Cases | Cases | Cases | Cases |
| MAF (%) | 0.3 | 2.3 | 3.1 | 0.3 | 0 | 26.5 | 31.1 | 0.3 | 0 | 0.3 | 0.3 | 0 | 0.3 | 24.5 | 23.5 | 0.3 |
| OR (95% CI) | NA | 0.65 (0.0.21, 1.71) | | NA | | 0.74 (0.50, 1.10) | | NA | | NA | NA | | NA | NA | NA | NA |
| P-value | NA | 0.51 | | 0.30 | | 0.12 | | 0.30 | | NA | 0.30 | | NA | NA | NA | NA |
|  |  |  | |  | |  | |  | |  |  | |  |  |  |  |
| EVS MAF (%)^c^ | NA | 2.3 | | 0.01 | | 25.4 | | NA | | NA | NA | | 0.06 | 29.8 | 27.2 | 0.01 |
| OR (95% CI) – cases vs. EVS | NA | 0.85 (0.30, 1.92) | | 23.65 (0.30, 1830.48) | | 1.13 (0.80, 1.58) | | NA | | NA | NA | | 5.34 (0.11, 48.12) | 0.81 (0.58, 1.13) | 0.85 (0.61, 1.19) | 26.39 (0.34, 2038.50) |
| P-value | NA | 0.84 | | 0.080 | | 0.50 | | NA | | NA | NA | | 0.20 | 0.22 | 0.37 | 0.072 |
|  |  |  | |  | |  | |  | |  |  | |  |  |  |  |
| 1000G MAF (%) | 0.08 | 1.0 | | NA | | 35.0 | | NA | | NA | NA | | 0.02 | 22.2 | 21.0 | 0.7 |
| OR (95% CI) – cases vs. 1000G | 4.05 (0.08, 41.30) | 1.98 (0.68, 4.71) | | NA | | 0.64 (0.45, 0.89) | | NA | | NA | NA | | 16.21 (0.21, 1261.59) | 1.31 (0.93, 1.83) | 1.26 (0.89, 1.77) | 0.49 (0.02, 2.95) |
| P-value | 0.26 | 0.14 | | NA | | 0.0077 | | NA | | NA | NA | | 0.11 | 0.11 | 0.17 | 0.72 |
|  |  |  | |  | |  | |  | |  |  | |  |  |  |  |
| PolyPhen-2 | NA | NA | | Benign | | Benign | | NA | | NA | Probably damaging | | NA | NA | NA | NA |
| SIFT | Tolerated | NA | | Tolerated | | Tolerated | | NA | | Tolerated | Damaging | | Tolerated | Tolerated | Tolerated | Tolerated |

^a^ cDNA reference (NM_015697.7) from NCBI gene was used to annotate the position of genomic DNA. ^b^ Accession number NP_056512.5 from NCBI protein were used to annotate the position of amino acid. ^c^ Data of European American population from Exome Variant Server. P-values result from Fisher’s exact test comparing the frequency of carriers of the minor allele between MSA patients and the separate groups of controls, EVS data, and 1000 Genomes data. Estimation of an odds ratio (and 95% confidence interval) is not possible when the rare allele is not observed in one of the comparison groups, and therefore NA is given for these quantities for three of the variants for which the rare allele was observed in MSA patients but not in controls. After applying a Bonferroni adjustment for multiple testing, p-values ≤0.01 (MSA patients vs. controls, 5 tests), ≤0.0071 (MSA patients vs. EVS data, 7 tests), and ≤0.0071 (MSA patients vs. 1000 Genomes data, 7 tests) were considered as statistically significant. Abbreviation: Cont, Control subjects; MAF, minor allele frequency; EVS, Exome Variant Server (<http://evs.gs.washington.edu/EVS/>); NA, not available; 1000G, 1000 Genomes Browser (<http://browser.1000genomes.org/>); OR, odds ratio; CI, confidence interval.
